# Interplay Between Reactive Oxygen Species and the Inflammasome Are Crucial for Restriction of *Neospora caninum* Replication

**DOI:** 10.3389/fcimb.2020.00243

**Published:** 2020-05-25

**Authors:** Caroline M. Mota, Djalma de S. Lima-Junior, Flávia Batista Ferreira França, Jhoan David Aguillón Torres, Patrício da Silva Cardoso Barros, Fernanda Maria Santiago, Joāo Santana Silva, José Roberto Mineo, Dario S. Zamboni, Tiago W. P. Mineo

**Affiliations:** ^1^Laboratory of Immunoparasitology “Dr. Mário Endsfeldz Camargo”, Institute of Biomedical Sciences, Universidade Federal de Uberlândia, Uberlândia, Brazil; ^2^Faculdade de Medicina de Ribeirão Preto, Universidade de São Paulo, São Paulo, Brazil

**Keywords:** *N. caninum*, ROS, inflammasome, macrophages, mice

## Abstract

*Neospora caninum* poses as a considerable threat to animal health and generates significant economic impact in livestock production worldwide. Here, we have investigated the mechanism that underlies the participation of the inflammasome complex and Reactive Oxygen Species (ROS) in the regulation of immune responses during *N. caninum* infection. For that purpose, we used *in vitro* (bone marrow derived macrophages) and *in vivo* mouse models of infection. Our results show that NLRP3 and NLRC4 receptors, alongside with ASC and Caspase-1, are required for proper activation of the inflammasome during *N. caninum* infection. As expected, the engagement of these pathways is crucial for IL-1α, IL-1β, and IL-18 production, as well as the induction of pyroptosis. Our results also show that *N. caninum* induces ROS production dependent of the inflammasome assembly, which in its turn also depends on MyD88/NF-κB-induced ROS to maintain its activation and, ultimately, lead to restriction of parasite replication.

## Introduction

*Neospora caninum* is an obligate intracellular parasite of the phylum Apicomplexa, that is able to infect different animal species although most commonly associated with bovine abortion worldwide (Horcajo et al., 2016).

The host protective immunity against *N. caninum* involves early production of the pro-inflammatory cytokine interleukin-12 (IL-12) by macrophages and dendritic cells (DCs), in response to recognition of pathogen-associated molecular patterns (PAMPs) and danger-associated molecular patterns (DAMPs) by Toll- like receptors (TLR) (Mineo et al., 2009, 2010). IL-12 stimulates natural killer (NK) cells, alongside with CD4^+^ and CD8^+^ T cells, to release interferon-γ (IFN-γ), which induces different killing mechanisms—as macrophage activation and reactive oxygen species (ROS) production. It has been previously suggested that parasite proliferation *in vivo*/*in vitro* is dependent on the absence or suppression of the cellular respiratory burst, and that the role of ROS in host defense against protozoa still deserves further assessment, since its parasiticidal mechanisms are still not completely known (Shrestha et al., 2006; Moreira-Souza et al., 2017; Li and Zhang, 2018).

In the last decade, a family of patterns recognition receptors (PRRs), called Nucleotide-binding Oligomerization Domain (NOD)-Like Receptors (NLRs) has emerged as an important innate immune sensor of protozoan parasites (Melo et al., 2011; Gurung and Kanneganti, 2016; Hakimi et al., 2017). NLRs are involved in the assembly of a cytosolic multi-protein complex called inflammasome, upon recognition of a ligand. The inactive caspase is recruited to this complex, in which it is cleaved. After proteolytic activation, Caspase-1/11 is able to cleave pro-IL-1β and pro-IL-18 cytokines into its active forms and may also result in a programmed form of cell death, named pyroptosis (Zamboni and Lima-Junior, 2015; Gurung and Kanneganti, 2016; Bierschenk et al., 2017; Kovacs and Miao, 2017). The activation of the inflammasome in response to infection by intracellular pathogens has recently gained attention of the scientific community. Research groups have linked mutations in this pathway to uncontrolled parasite growth (Fink and Cookson, 2006; Riteau et al., 2016; Wang et al., 2017).

In this study, we assessed the interplay between ROS production and the inflammasome activation during *N. caninum* infection. Our results suggest that the engagement of the NLRP3 and NLRC4 inflammasomes have a crucial role in the restriction of *N. caninum* replication. Notably, inflammasome activation by *N. caninum* is independent of previous cell priming and triggers the production of ROS, a major host defense mechanism against intracellular parasites. In addition, we also show in the context of the infection that oxidative stress directly activates the inflammasome to control the infection.

## Materials and Methods

### Parasites and Antigens

NIH/3T3 (ATCC® CRL-1658™) were cultured in RPMI-1640 medium supplemented with 10% heat- inactivated fetal bovine serum (FBS), 100 U/ml penicillin/streptomycin, and cells were maintained in an incubator at 37°C in a humidified atmosphere of 5% CO_2_. The cells were treated with Plasmocin^TM^ (InvivoGen, USA) for 2 weeks before parasite infection and screened by PCR for *Mycoplasma* spp., in order to avoid bacterial contamination in parasite stocks. Parasites were maintained *in vitro* by serial passages on NIH/3T3 monolayers, cultured in RPMI 1640 medium supplemented with 2 mM glutamine, 100 U/mL penicillin, 100 μg/mL streptomycin and 250 ng Amphotericin B (Gibco), at 37°C in 5% CO_2_ atmosphere. Briefly, tachyzoites were harvested by scraping off the cell monolayer after 48–72 h of infection containing mainly intracellular parasites (at least 90%), passed through a 26-gauge needle to lyse any remaining intact host cell, and centrifuged at low speed (45 × g) for 1 min at 4°C to remove host cell debris. The supernatant containing parasite suspension was collected and pelleted (800 × g, 10 min, 4°C). Tachyzoites were counted in hemocytometry chamber using 0.4% Trypan blue vital staining and immediately used for the experiments. Parasites of the Nc-Liverpool isolate of *N. caninum* (NcLiv, Barber et al., 1995) were used in all experimental settings. Occasionally, infections with the *N. caninum* isolate 1 (Nc-1, Dubey et al., 1988) were also included in the experiments.

In order to test the effects of viability and distinct antigenic fractions in the proposed context, we also exposed macrophages to fixed or heat attenuated parasites, as well as to *Neospora* lysate antigens (NLA) and excreted-secreted antigens (ESA), produced according to previous description (Ribeiro et al., 2009; Mota et al., 2016). Parasite suspensions of freshly lysed tachyzoites (~10^8^) were washed at least twice in phosphate buffered saline (PBS, pH 7.2) for antigen preparation. For NLA, the parasites were lysed by 10 freeze-thaw cycles followed by ultrasound disruption on ice, in the presence of protease inhibitors (Complete, Roche). After centrifugation (10,000 × g, 30 min, 4°C), the supernatant was collected, filtered on 0.22 μm membranes and its protein concentration determined by the Bradford method. For ESA, the parasite pellet was resuspended in Hank's saline solution (30 min, 37°C), with gentile agitation. After centrifugation (800 × g, 10 min, 4°C) and the supernatant was collected and centrifuged again (10,000 x g, 30 min, 4°C) to eliminate any insoluble material from the preparation. The supernatant was then filtered (0.22 μm) and its protein concentration determined by Bradford method. NLA and ESA were aliquoted and stored at −20°C until being used in the experiments. Fixed tachyzoites were obtained by the incubation with 4% formaldehyde (30 min, at room temperature). The fixed parasites were then washed and resuspended in PBS. Heat attenuated tachyzoites were obtained by incubation of the parasite suspension at 56°C for 50 min. Fixed and heat inactivated tachyzoites were counted and immediately used in the experiments.

### Mice

WT C57BL/6 mice (JAX 000664), along with genetically deficient littermates in Caspase-1 and Caspase-11 (*Casp-1/11*^−/−^, Kuida et al., 1995); *Casp-1/11*^−/−^ complemented with Caspase-1 (*Casp-1*^−/−^; Kayagaki et al., 2011); NACHT, LRR and PYD domains-containing protein 3 (*Nlrp3*^−/−^; Mariathasan et al., 2006); NLR family CARD domain-containing protein 4 (*Nlrc4*^−/−^; Mariathasan et al., 2004); Apoptosis-associated speck-like protein containing a CARD (*Asc*^−/−^; Mariathasan et al., 2004), Myeloid differentiation primary response 88 protein (*Myd88*^−/−^; Adachi et al., 1998), NADPH oxidase 2 gp91^phox^ subunit (*Nox2*^−/−^; Pollock et al., 1995), and IL-1 receptor (*Il-1r*^−/−^; Glaccum et al., 1997) were bred under specific pathogen-free conditions at the animal facilities of University of São Paulo (FMRP/USP) and Federal University of Uberlândia (REBIR/UFU). The animals were supplied with 6–10 weeks of age and maintained at REBIR/UFU in individual cages, under controlled conditions (12 h light and 12 h dark cycle, controlled temperature of 22 ± 2°C), and received food and water *ad libitum*. All protocols involving mice were previously approved by the institution's animal research ethics committee (Comitê de Etica na Utilização de Animais da Universidade Federal de Uberlândia—CEUA/UFU), under protocol number 109/16, and were carried out in accordance with the recommendations in the International Guiding Principles for Biomedical Research Involving Animals of the International Council for Laboratory Animal Science (ICLAS), countersigned by the Brazilian National Council for the Control of Animal Experimentation (Conselho Nacional de Controle de Experimentação Animal, CONCEA; https://olaw.nih.gov/sites/default/files/Guiding_Principles_2012.pdf). REBIR/UFU is accredited by the National Commissions in Animal Experimentation (CONCEA, CIAEP 01.0105.2014) and Biosecurity (CTNBio, CQB 163/02).

### Bone Marrow-Derived Macrophages (BMDM)

BMDMs were obtained from WT and genetically deficient mice after a 6-day differentiation in L929-conditioned media, as previously described (Mota et al., 2016). Briefly, stem cells were cultured on 10 cm-diameter polystyrene plates, for 6 days in RPMI- 1640 medium, containing HEPES 15 mM, 2 g of sodium bicarbonate/L, 1 mM l-glutamine, supplemented with 20% heat-inactivated FCS and 30% cell-conditioned medium, obtained from the supernatant of confluent L929 cells. Differentiated BMDMs were removed from the plates by vigorous pipetting of ice-cold PBS. Cells were counted in hemocytometry chamber using 0.4% Trypan blue vital staining and set for experiments at 2 ×10^5^ and 1 x 10^6^ cells per well in 96-well and 24-well plates, respectively.

### *In vitro* Stimulation Assays

BMDMs were plated at 2 ×10^5^ cells per well (1 ×10^6^/mL) in 96-well plates and stimulated with live, fixed or heat attenuated *N. caninum* tachyzoites at multiplicity of infection (MOI) of 0.5 (1 parasite per 2 cells) or antigen of NLA and ESA, in kinetics up to or at 18 h endpoint. This experimental setup was chosen after preliminary assays that showed higher production of IL-1β in MOI 0.5, compared to MOIs 1 and 3 (Supplementary Figure 1A). BMDM lysis was perceptible in the higher MOIs tested at 18 h of infection, as well as in any other dose for longer periods of incubation. For some assays, the cells were pre-treated with 500 ng/mL *Salmonella thypimurium* LPS (TLR4/Caspase-11 agonist, Sigma), 5 μM Caffeic acid phenethyl ester (CAPE, NF-κB inhibitor, Sigma), 10 μg/mL Tanshinone (AP-1 inhibitor, Sigma), 25–200 μM KCl (Sigma) or 25–200 μM NaCl (Sigma) for 3 h, and for 1 h with 100 μM N-acetyl-cysteine (NAC, ROS scavenger, Sigma), 2.5 μM ATP (NLRP3 inducer, Invivogen) or 5 μM Nigericin (NLRP3 agonist, Invivogen). The wells were washed with fresh media to remove the drugs before the addition of *N. caninum* tachyzoites.

### *In vivo* Infection Assays

WT C57BL/6, along with CASP1/11^−/−^, with 6 to 8 weeks old, were infected (*n* = 5/group) with sublethal doses (1 ×10^6^) of NcLiv tachyzoites per animal (i.p.). After 3 days post infection, mice were euthanized by cervical dislocation, and their peritoneal cells were removed by washing the cavities with ice-cold PBS. The peritoneal cells were used for measurement of Caspase-1/11 activity, ROS, pyroptosis and quantification of parasite burden. Liver fragments were also collected for the quantification of parasite burden by qPCR. Additional groups of mice were similarly infected with NcLiv and euthanized after 30 days, had their brains removed for parasite burden quantification.

### Endogenous Caspase-1 Staining Using FAM-YVAD-Fluoromethylketone (FMK)

BMDMs and peritoneal cells were plated at 2 ×10^5^ cells per well (1 ×10^6^/mL) in 96-well black plates with clear bottom (Costar) or at 1 ×10^6^ cells/well in 24-well plates. After 18 h of infection, the cells were stained for 1 h with FAM-YVAD-FMK as recommended by the manufacturer's instructions (Immunochemistry Technologies). Active Caspase-1/11 was then measured by a plate fluorimeter (M2e, Molecular Devices), analyzed in a fluorescent microscope (EVOS fl, ThermoFisher), or by flow cytometry (FACSCantoII, BD). For the flow cytometry analysis, we considered the percentage of positive cells and the mean fluorescence intensity (MFI). Both parameters were used to calculate the factor iMFI, which consists of the multiplication of between the number of positive cells by MFI (Darrah et al., 2007).

### Western Blot

A total of 4 ×10^6^ BMDMs were seeded per well in 6-well plates, infected with NcLiv tachyzoites for 18 h. The supernatants were collected and cells were lysed in RIPA buffer (10 mM Tris-HCl, pH 7.4, 1 mM EDTA, 150 mM NaCl, 1% Nonidet P-40, 1% deoxycholate, and 0.1% SDS) supplemented with protease inhibitors cocktail (Roche). Lysates and supernatants were boiled in Laemmli buffer, resolved by SDS-PAGE 12%, and transferred (Semidry Transfer Cell, Bio-Rad) to 0.22-μm nitrocellulose membranes (GE Healthcare). The rabbit anti–IL-1β/IL-1F2 polyclonal antibodies (Novus; 1:1000), and species-specific horseradish peroxidase-conjugated secondary antibodies (R&D Systems; 1:1000) were used for antigen detection. The blot was incubated with ECL substrate (Promega) and chemiluminescence was detected using an imaging system with dedicated software (ChemiDoc XRS, Bio-Rad).

### Cytokine ELISAs

IL-1β, IL-1α, and IL-18 were measured by ELISA using commercial kits, according to the manufacturer's instructions (BD Biosciences and R&D Systems).

### Evaluation of Cytotoxicity by Lactate Dehydrogenase (LDH) Release

Supernatants obtained from 2 ×10^5^ BMDMs per well (1 ×10^6^/mL) in 96-well plate of infected and naïve cells were collected, and the activity of released LDH was measured using colorimetric assays, according to manufacturer's instructions (Thermo Scientific). Data are expressed as a percentage of LDH release induced in BMDMs by Triton-X100 [(sample OD x 100)/Triton-X100 OD].

### Membrane Pore Formation Assay

The kinetics of pore formation were assessed by quantifying the uptake of propidium iodide (PI) into infected cells (Cunha et al., 2017). BMDMs were plated at 2 ×10^5^ cells per well (1 ×10^6^/mL) in 96-well black plate with clear bottom (Costar). Before infection, BMDM media was replenished with 2% SFB RPMI without phenol red, NaHCO_3_ (0.038 g/mL), and PI (6 μg/mL). Infected BMDMs were maintained at 37°C with 5% CO_2_; PI was excited at 538 nm, and fluorescence emissions were read at 617 nm every 5 min with a plate fluorimeter (M2e, Molecular Devices) or analyzed in a fluorescent microscope (EVOS fl, ThermoFisher). The peritoneal cells extracted from *in vivo* infections were seeded at 2x10^5^ cells per well (1 ×10^6^/mL) in 96-well black plate with clear bottom (Costar) and stained for 3 h with PI at 37°C and 5% CO_2_ before the measurements were taken in a plate fluorimeter.

### ROS Production

BMDMs and peritoneal cells were plated at 2 ×10^5^ cells per well (1 ×10^6^/mL) in 96-well black plates with clear bottom (Costar). After 3 or 18 h of infection, the cells were stained for 30 min with 1 μM DHCFDA (carboxymethyl-H2-dichlorofluorescein diacetate, Molecular Probes) at 37°C and 5% CO_2_. The reactions were read in a plate fluorimeter (M2e, Molecular Devices) or analyzed in a fluorescent microscope (EVOS fl, ThermoFisher).

### Determination of Parasite Burden

*N. caninum* tachyzoites in the infected cell cultures was determined by fluorescent ester-based probe as previously described (Mota et al., 2014), parasites were stained with 5μM/mL of DDAO-SE (Thermo Scientific). After 10 min at 37°C, the tachyzoites were washed with 10 mL of RPMI-1640 with 10% FCS and centrifuged at 800 × g for 10 min at 4°C. Viable tachyzoites were determined with the Trypan blue exclusion test and used to infect BMDM. After 18 h, the infected cell monolayer was harvested and read in a flow cytometer (FACSCantoII, Becton, Dickinson and Company—BD, Franklin Lakes, NJ, USA) with at least 50,000 events acquired per tube.

Liver and brain parasite burden was determined by quantitative real-time PCR as previously described (Ribeiro et al., 2009), by the use of primer pairs (sense 3′ -GCTGAACACCGTATGTCGTAAA-5; antisense 3′-AGAGGAATGCCACATAGAAGC-5) to detect the *N. caninum* Nc-5 sequence. DNA extraction was performed from 20 mg of murine tissues (Genomic DNA kit, Promega Co., USA) and parasite loads were calculated by interpolation from a standard curve of NcLiv tachyzoite DNA included in each run. As negative control, DNA obtained from liver or brain tissues of non-infected mice was analyzed in parallel. The amplification, data collection and analysis were performed with a real-time PCR thermal cycler (StepOne Plus, Life, Thermo Scientific) using the SYBR green system (PowerUpTM SYBR Green Master Mix, ThermoFisher). In addition, parasitism of the peritoneum was determined after 3 days of infection, the animals were euthanized, and their intraperitoneal cavity washed with 1 mL of PBS and saved for counting parasites by two independent observers by using Trypan blue.

### Statistical Analysis

Statistical analysis was carried out using GraphPad Prism 8.0 (GraphPad Software Inc., La Jolla, CA, USA). After passed to the normality tests, values were expressed as mean ± standard error, and analyzed by one-way ANOVA test, followed by Bonferroni *post hoc* test for comparison among the groups or Student *t*-test was used for comparison between two groups. Values of *P* <0.05 were considered statistically significant. Each experiment was independently conducted at least two times, and each condition was analyzed in triplicates, at least.

## Results

### *Neospora caninum* Induces Activation of the NLRP3 and NLRC4 Inflammasome Complexes, Dependent of MyD88 and Caspase-1

**Figure 1:**
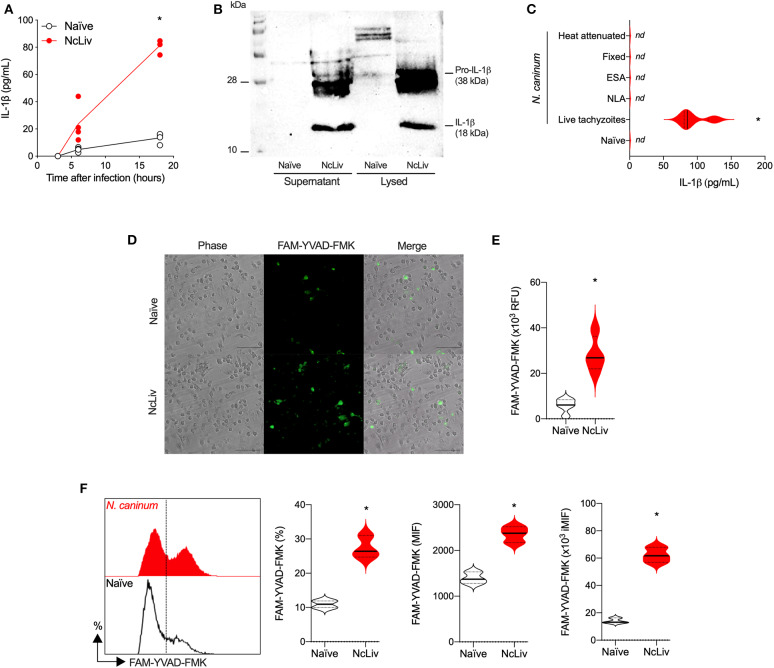
Without previous priming, the inflammasome complex is activated in response to *Neospora caninum*. Bone marrow-derived macrophages (BMDMs) were infected with *N. caninum* tachyzoites (NcLiv; MOI 0.5). Kinetics of IL-1β production was measured by ELISA **(A)** or detected by western blotting in the supernatant and lysed cells (18 h, **B**). IL-1β secretion was measured by ELISA in BMDMs exposed to live, fixed, or heat inactivated tachyzoites, or stimulated (10 μg/mL) with *Neospora* lysate antigens (NLA) or excreted secreted antigens (ESA) **(C)**. Caspase-1/11 (CASP-1/11) activity was detected in BMDMs infected with NcLiv tachyzoites after 18 h (MOI 0.5) using the fluorescent FAM-YVAD-FMK probe through microscopy **(D)** in a plate reader **(E)** or through flow cytometry—represented by histogram, percentage of positive cells and expression levels (MIF; iMIF = % of positive cells X mean intensity of fluorescence of the population) **(F)**. Values are representative of at least two independent experiments and each condition was conducted at least in triplicates. Values indicating mean ± SEM of cytokine levels in relation the standard curve and CASP-1/11 activity (mean fluorescence intensity—MIF or in relative fluorescence units – RFU) (*P <0.05; ANOVA with the Bonferroni multiple comparison *post-hoc* test or *t*-test between naïve and *N. caninum* infected BMDMs).

The NLRP3 inflammasome has been described to become activated in response to several intracellular pathogens, including *N. caninum* (Zamboni and Lima-Junior, 2015; Riteau et al., 2016; Wang et al., 2017, 2019). First, in order to validate the model within our experimental conditions, we ran a set of experiments using BMDMs exposed to *N. caninum* to determine the kinetics of IL-1β production in macrophages during infection. For that purpose, WT BMDMs were infected with tachyzoites at multiplicity of infection (MOI; cell:parasite ratio) of 0.5 and IL-1β production was measured in different times of infection (3, 6, and 18 h). This MOI was chosen to ensure the measurement of higher levels of active IL-1β by ELISA (Supplementary Figure 1A), whereas longer periods of incubation caused significant host cell lysis and consequent release of the pro-form of the cytokine into the supernatant (data not shown). Also, in this experimental scenario, we estimate that ~50% of the BMDMs were infected after 18 h (Supplementary Figure 1B). As seen in Figure 1A, there is a notable buildup in the concentration of IL-1β as the infection progressed. We also looked for active/secreted IL-1β as a product of the enzyme's activity. We found that naïve WT BMDMs did not produce measurable levels of pro- (38 kDa) or active (18 kDa) IL-1β, whereas both forms of the cytokine were robustly detected after infection with live *N. caninum* tachyzoites (Figure 1B).

Next, we sought to determine whether IL-1β production by BMDMs exposed to *N. caninum* required actual infection mechanisms by the parasite or if different antigenic fractions were capable of inducing the release of the cytokine. As shown in Figure 1C, live tachyzoites were the only stimuli able to induce IL-1β production by BMDMs. In order to measure Caspase-1/11 (CASP-1/11) activity, we used the fluorescent probe (FAM-YVAD-FMK), which irreversibly binds to active CASP-1/11 (20 kDa). As expected, we detected a higher percentage of CASP-1/11+ BMDMs exposed to live *N. caninum* tachyzoites by microscopy (Figure 1D), increased fluorescent signal in a plate reader (Figure 1E), and increased percentage and expression (MFI) by flow cytometry (Figure 1F), that from here on will be expressed as the correlation of both values (iMFI). Noteworthy, we also tested whether these phenotypes would be conserved in different *N. caninum* isolates, directly comparing NcLiv with Nc-1. As it may be observed in Supplementary Figure 2, WT BMDMs infected with either isolates produced similar amounts of IL-1β (Supplementary Figure 2A) and had almost identical percentages of CASP-1/11 positive cells (Supplementary Figure 2B).

**Figure 2:**
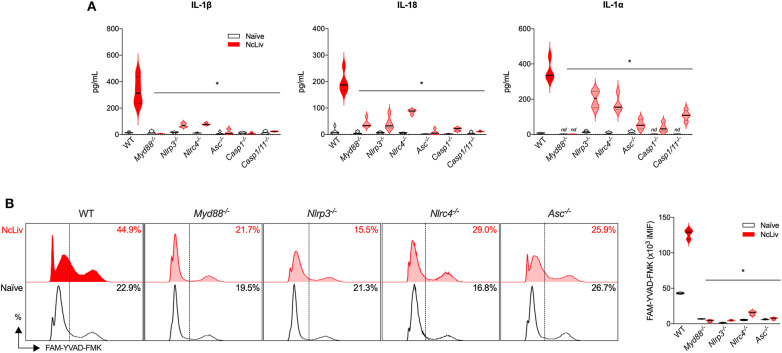
*N. caninum* promotes inflammasome activation through NLRP3 and NLRC4 receptors, in a MyD88-dependent manner. BMDMs obtained from WT, *Myd88*^−/−^, *Nlrp3*^−/−^, *Nlrc4*^−/−^, *Asc*^−/−^, *Caspase-1*^−/−^ and *Caspase-1/11*^−/−^ mice were infected for 18 h with *N. caninum* tachyzoites (NcLiv; MOI 0.5). IL-1β, IL-18, and IL-1α were measured by ELISA **(A)**. Caspase-1/11 activity was assessed by flow cytometry, using the fluorescent probe FAM-YVAD-FMK. These results were represented in histograms indicating the % of FAM-YVAD-FMK-positive cells and expression levels (iMIF = % of positive cells X mean intensity of fluorescence of the population) **(B)**. Values are representative of at least two independent experiments and each condition was conducted at least in triplicates. Values indicating mean ± SEM of cytokine levels in relation the standard curve and CASP-1/11 activity (*P <0.05; ANOVA with the Bonferroni multiple comparison *post-hoc* test between WT and genetically deficient BMDMs infected with *N. caninum*).

Next, we sought to further investigate the immune signaling involved in the inflammasome activation induced by *N. caninum*. For that purpose, we verified whether BMDMs deficient in components of the pathway were able to release IL-1β, IL-1α, and IL-18 in the same manner as WT cells in response to the infection by live tachyzoites (Figure 2A). We also checked which of the tested genes would negatively influence the upregulation of CASP-1/11 in BMDMs cocultured with NcLiv (Figure 2B). The experiments were performed alongside with cells deficient in the major TLR adaptor protein MyD88, known to participate in the early responses against *N. caninum* (Mineo et al., 2009, 2010), contributes to the production of pro-IL-1β and IL-1 receptor signaling (Dinarello, 2009). Genetically deficient cells failed (partially or completely) to induce CASP-1/11 and the consequent release of the cleaved cytokines into the supernatant in response to the infection by NcLiv tachyzoites. Noteworthy, NLRC4 is also required for full inflammasome activation, in addition to the already described NLRP3 pathway (Wang et al., 2017). In addition, two other relevant results derived from this experimental setup are significant: (1) Caspase-11 has no or limited role in the cleavage of the cytokines induced by the infection with live tachyzoites, as single knockout BMDMs to Caspase-1 produced similar levels of the active cytokines if compared to the double knockout cells (*Casp-1/11*^−/−^); (2) MyD88, an adaptor protein for most TLRs and also the IL-1 receptor, is a crucial component of the Inflammasome activation.

**Figure 3:**
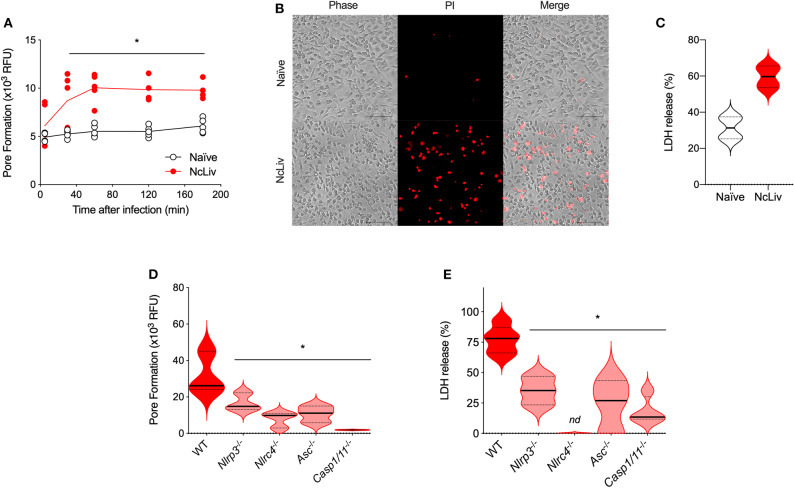
*N. caninum* infection induces NLRP3- and NLRC4-dependent pyroptosis in macrophages. BMDMs were infected with *N. caninum* tachyzoites (NcLiv; MOI 0.5). Kinetics of pore formation was measured using propidium iodide (PI) in a plate reader **(A)** and visualized by microscopy after 3 h of infection **(B)**. Lactate dehydrogenase (LDH) release was measured after 18 h of infection using colorimetric assays and expressed as the percentage of LDH release, compared to the assay's positive control (BMDMs in Triton-X100, **C**). BMDMs obtained from WT, *Nlrp3*^−/−^, *Nlrc4*^−/−^, *Asc*^−/−^, and *Caspase-1/11*^−/−^ mice were infected with NcLiv (MOI 0.5). Pore formation was measured after 3 h of infection by PI incorporation **(D)** and LDH release was measured after 18 h **(E)**. Values are representative of at least two independent experiments and each condition was conducted at least in triplicates. Values indicating mean ± SEM for PI incorporation (relative fluorescence units—RFU) and percentage of LDH release (*P <0.05; ANOVA with the Bonferroni multiple comparison *post-hoc* test between WT and genetically deficient BMDMs infected with *N. caninum* or *t*-test between naïve and *N. caninum* infected BMDMs).

Another feature of the inflammasome is the cell death triggered in response to intracellular pathogens, induced by pore formation in the cellular membranes, also called pyroptosis (Fink and Cookson, 2006). To investigate the pore-forming ability of BMDMs in response to *N. caninum*, we first performed experiments using naïve WT BMDMs exposed to live NcLiv tachyzoites and evaluated the loss of membrane integrity by propidium iodide (PI) incorporation and lactate dehydrogenase (LDH) release after 18 h. We found that *N. caninum* triggered cell death that peaked between 60 and 120 min of infection, a phenomenon detected by the increased PI incorporation (Figures 3A,B) and LDH release (Figure 3C), compared to uninfected cells. In addition, to determine the components required for inflammasome-induced cell death during the infection, we assessed whether genetically deficient macrophages would present impaired pyroptosis after exposure to live tachyzoites. We observed that genetic disruption of each of the tested components of the pathway led to decreased PI incorporation (Figure 3D) and LDH release (Figure 3E), especially in *Nlrc4*^−/−^ and *Casp-1/11*^−/−^ BMDMs, in comparison with WT BMDMs.

### The Interplay Between ROS and the Inflammasome in Response to *N. caninum*

**Figure 4:**
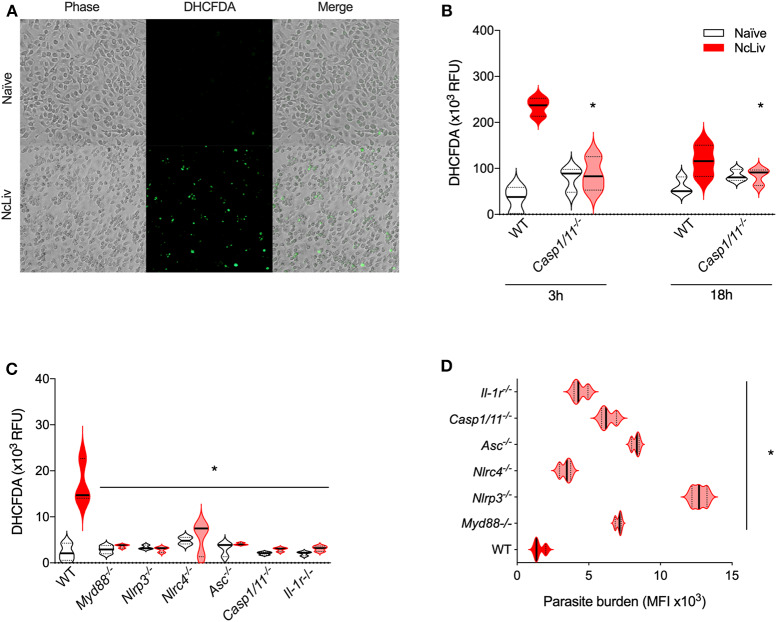
The inflammasome complex is required for ROS production and consequent restriction of *N. caninum* replication. ROS production was assessed by the fluorescent DHCFDA probe, as observed by microscopy of naïve BMDMs and cells infected with *N. caninum* tachyzoites (NcLiv; MOI 0.5) for 18 h **(A)**. BMDMs obtained from WT and *Caspase-1/11*^−/−^ mice were infected for 3 and 18 h with NcLiv (MOI 0.5). ROS production was measured in a plate reader **(B)**. BMDMs obtained from WT, *Myd88*^−/−^, *Nlrp3*^−/−^, *Nlrc4*^−/−^, *Asc*^−/−^, *Caspase-1*^−/−^, and *Caspase-1/11*^−/−^ mice were infected for 18 h with NcLiv (MOI 0.5) and ROS production was measured in a plate reader **(C)**. Parasite burden was assessed using DDAO-SE (fluorescent ester-based probe) stained parasites and measured by flow cytometry, as results were represented in expression levels (MIF) **(D)**. Values are representative of at least two independent experiments and each condition was conducted at least in triplicates. Values indicating mean ± SEM of fluorescence levels (mean fluorescence intensity—MIF or in relative fluorescence units—RFU) (*P <0.05; ANOVA with the Bonferroni multiple comparison *post-hoc* test between infected WT and genetically deficient BMDMs or *t*-test between infected WT and *Casp1/11*^−/−^ BMDMs).

One of the primary mechanisms underlying host resistance against intracellular pathogen replication is the production of reactive oxygen species (ROS). It is known that ROS and its signaling pathway are involved in the resistance against *N. caninum* infection (da Silva et al., 2017). Therefore, to check the interplay between inflammasome complex activation and ROS production induced by *N. caninum*, we measured ROS in BMDMs exposed to live NcLiv tachyzoites using a fluorescent probe (DHCFDA, Figure 4A). First, we quantified ROS production in WT and *Casp-1/11*^−/−^ BMDMs exposed to live NcLiv tachyzoites, after 3 and 18 h of incubation. The results indicated a sharp ROS production in WT macrophages especially during at 3 h of infection, while a marked decreased of the relative fluorescence was perceived in *Casp-1/11*^−/−^ infected BMDMs in both time of 3 and 18 h after infection (Figure 4B). In addition, we evaluated whether other inflammasome components would be crucial to control *N. caninum* replication. Therefore, we performed *in vitro* parasite load assays using BMDMs infected with live pre-stained *N. caninum* tachyzoites, analyzed by flow cytometry, along with paired measurement of ROS by the cells. We found that the insufficient amounts of ROS produced by genetically deficient BMDMs (Figure 4C) were directly correlated to their permissiveness to the replication of parasites (Figure 4D)—especially regarding *Nlrp3*^−/−^ macrophages—if compared to WT cells.

**Figure 5:**
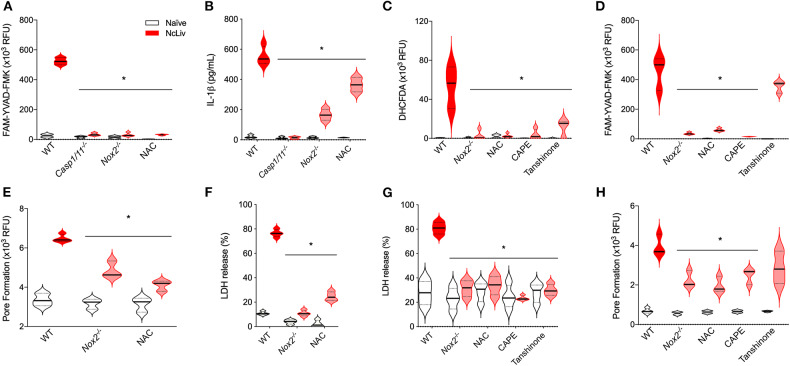
ROS production is required for the *Neospora caninum*-induced inflammasome activation in a NF-κB-dependent manner. BMDMs obtained from WT, *Nox2*^−/−^ and *Caspase-1/11*^−/−^ mice or WT treated for 1 h with N-acetyl-cysteine (NAC, ROS scavenger), and 3 h with 5 μM Caffeic acid phenethyl ester (CAPE, NF-κB inhibitor) or 10 μg/mL Tanshinone (AP-1 inhibitor), were infected with *N. caninum* tachyzoites (NcLiv; MOI 0.5). Caspase-1/11 (CASP-1/11) activity was measured in BMDMs after 18 h of infection using the fluorescent FAM-YVAD-FMK probe in a plate reader **(A,D)** and IL-1β production was measured in the supernatant by ELISA **(B)**. ROS production was measured after 18 h infection by fluorescent signal in a plate reader using fluorescent DHCFDA probe **(C)**. Pore formation was measured after 3 h of infection using propidium iodide (PI) incorporation in a plate reader **(E,H)** and Lactate dehydrogenase (LDH) release was measured after 18 h of infection using colorimetric assays and expressed as a percentage of LDH release compared to the assay's positive control (BMDMs in Triton-X100; **F,G**). Values are representative of at least two independent experiments and each condition was conducted at least in triplicates. Values indicating mean ± SEM of fluorescence levels (relative fluorescence units—RFU) and percentage of LDH release (*P <0.05; ANOVA with the Bonferroni multiple comparison *post-hoc* test between WT, treated and genetically deficient BMDMs, infected with *N. caninum*).

On the other hand, we also assessed whether the oxidative stress could interfere with inflammasome complex activation in response to *N. caninum*, through FAM-YVAD-FMK probe reactivity (Figure 5A) and IL-1β secretion (Figure 5B) in *Casp-1/11*^−/−^, *Nox2*^−/−^, and WT BMDMs treated with N-acetyl-cysteine (NAC), a ROS scavenger. It was striking to us that, in the absence of ROS signaling and production, the inflammasome activation was severely inhibited, while IL-1β levels presented a marked decrease. In addition, we sought to determine the host's transcription factors involved in the interplay between inflammasome complex formation and ROS production during *N. caninum* infection. NF-κB and AP-1 have been shown to trigger the ROS pathway during infections (Mazière et al., 1999; Huang et al., 2016). Indeed, we found that the ROS production and inflammasome activation by *N. caninum* was dependent on NF-κB, while partially dependent of the AP-1 pathway, in experiments where we measured the expression of ROS (Figure 5C) and CASP-1/11 (Figure 5D), in *N. caninum* infected WT BMDMs treated with NAC, CAPE (NF-κB inhibitor) or Tanshinone (AP-1 inhibitor), alongside with *Nox2*^−/−^ cells. We also investigated whether the oxidative stress could interfere in the cell death by pyroptosis in response to *N. caninum*, through PI incorporation (Figure 5E) and LDH release (Figure 5F) in *Nox2*^−/−^ and WT BMDMs, treated or not with NAC. It was also notable that, in the absence of ROS, pyroptosis was inhibited as demonstrated by significantly decreased pore formation and LDH release. Furthermore, we observed that pyroptosis induced by live tachyzoites was completely dependent of NF-κB and partially dependent of AP-1, in experiments using LDH release (Figure 5G) and PI incorporation (Figure 5H) as readouts, observing NcLiv-infected WT BMDMs. treated or not with NAC, CAPE (NF-κB inhibitor) or Tanshinone (AP-1 inhibitor), alongside with *Nox2*^−/−^ cells.

**Figure 6:**
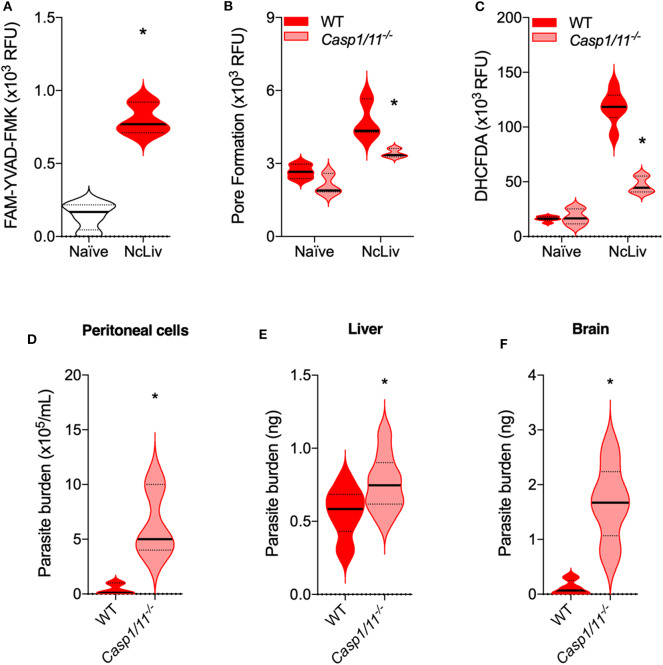
The absence of Caspase-1/11 leads to decreased pyroptosis and ROS production, while mice fail to properly restrict *N. caninum* replication *in vivo*. C57BL/6 WT and *Caspase-1/11*^−/−^ mice (*n* = 5/group) were infected with 1 ×10^6^ tachyzoites of *N. caninum* tachyzoites (NcLiv) and euthanatized after 3 days for the collection of peritoneal cavity cells and liver fragments, or after 30 days for the collection of brain fragments. Peritoneal cells were assessed in a plate reader for Caspase-1/11 (CASP-1/11) activity, measured by the fluorescent FAM-YVAD-FMK probe **(A)**; pore formation or pyroptosis, quantified by propidium iodide (PI) incorporation **(B)**; and ROS production, measured by the fluorescent DHCFDA probe **(C)**. Peritoneal parasite burden was determined counting the extracellular parasites in a hemocytometer **(D)**. Parasite burden in liver **(E)** and brain **(F)** fragments were measured by quantitative real-time PCR, and parasite loads were calculated by interpolation from a standard curve of NcLiv tachyzoite DNA **(E)**. Values are representative of two independent experiments and indicate mean ± SEM of fluorescence levels (relative fluorescence units—RFU) and parasite burden (*P <0.05; *t*-test between infected WT and *Casp1/11*^−/−^ mice).

In order to investigate whether the interplay between ROS and the Inflammasome during *N. caninum* infection was reproducible *in vivo*, we performed experiments to check the immune responses and parasite burden of WT and *Casp-1/11*^−/−^ mice, after 3 and 30 days of infection. We first analyzed peritoneal cells after the 3 days of *in vivo* infection, and established that there is an increase in FAM-YVAD-FMK probe reactivity (Figure 6A) in peritoneal cells of WT mice, as well as a decrease in PI incorporation in cells derived from *Casp-1/11*^−/−^ mice (Figure 6B). Also, within the same context, we assessed whether the intact inflammasome pathway would be crucial to control *N. caninum* replication and ROS production *in vivo*. We found that *Casp-1/11*^−/−^ mice presented a pronounced decrease in ROS production during the acute infection (Figure 6C) and, in association, it was detected a six-fold increase of parasite burden in the genetically deficient mice (Figure 6D). Significant increments in parasite burden of *Casp-1/11*^−/−^ mice were also observed in liver (3 days of infection, Figure 6E) and brain (30 days of infection, Figure 6F) samples.

## Discussion

Innate immunity plays an important role in protection and pathogenesis of protozoan infections, including *N. caninum*. MyD88-dependent TLR signaling has been reported to play a key role in parasite recognition and induction of suitable immune response against this parasite (Mineo et al., 2009, 2010; Beiting et al., 2014; Gurung and Kanneganti, 2016). However, activation of MyD88-independent TLR pathways, such as the TRIF-dependent TLR3 signaling, are also important for activation of the immune response against *N. caninum*, as potent inducers of type I interferons (Beiting et al., 2014; Miranda et al., 2019). In parallel, NLRs have also emerged as important components of the innate immune system, due to its ability to recognize and eliminate intracellular parasites (Zamboni and Lima-Junior, 2015; Coutermarsh-Ott et al., 2016). Recent studies have reported that NLR-mediated host immune responses contribute to *N. caninum* elimination and pathogenesis of neosporosis (Davoli-Ferreira et al., 2016; Wang et al., 2017, 2018, 2019). In this study, we identified the NLRP3 and NLRC4 inflammasomes as critical innate immune components during *N. caninum* infection in macrophages that, along with MyD88-dependent TLR responses, coordinate immune restriction mechanisms against the parasite replication.

The inflammasome activation requires a priming step, typically provided by LPS pre-treatment as first signal in different experimental setups, which induces the expression of biologically inactive precursors (pro-CASP-1, pro-IL-18, and pro-IL-1β) and sensor molecules that need to be activated via auto-proteolysis processing. Consequently, the activation that leads to the formation of a cytosolic multi-protein signaling complex, the inflammasome, requires two distinct signals from pathogen-associated molecular patterns (PAMPs) or host-derived danger-associated molecular pattern (DAMPs) (Afonina et al., 2015; Zhu and Kanneganti, 2017). In our study, we demonstrated that *N. caninum* is able to induce both signals by itself in BMDMs, in order to fully active the inflammasome. To further demonstrate that capability, we also ran experiments with known agonists of the inflammasome (LPS and ATP) side-by-side with NcLiv tachyzoites (Supplementary Figure 3). These processes involve the regulation of the active cytokines IL-18, IL-1α, and IL-1β. Thus, NLRP3 and NLRC4 receptors—along with ASC and CASP-1/11—are crucial for secretion of active factors that culminate in ROS production, that will ultimately restrict *N. caninum* growth. While the demonstration that the NLRC4 Inflammasome participates in the context of *N. caninum* infection is entirely new, the activation of the NLRP3 inflammasome has been previously demonstrated in mouse macrophages and bovine monocytes (Wang et al., 2017, 2019), although the authors showed a similar mechanism in LPS-primed BMDMs (Wang et al., 2018), not in naïve cells as we have shown in this work.

In addition, the activation of inflammasome-associated inflammatory caspases drives cleavage of the pro-pyroptotic factor gasdermin D to form pores on the host cell, causing membrane permeabilization and consequent pyroptosis. This process is required to restrict the replication of intracellular pathogens by eliminating the infected cell and removing the protective niche of the pathogen, while simultaneously elicits an inflammatory response (Cunha et al., 2015; Kovacs and Miao, 2017; Man et al., 2017). Based on previous work, pyroptosis has been shown to be regulated by CASP-1/11-dependent or -independent mechanisms. Caspase-1-independent pyroptosis is executed by human Caspase-4, human Caspase-5, or mouse Caspase-11. This alternative pyroptosis pathway presents a similar phenotype to that induced by CASP-1 alone, while also leads to the release of IL-1β and IL-18 (Man et al., 2017). Our data reveal a partial role for NLRP3-inflammasome in this context, while pore formation and consequent pyroptosis triggered by *N. caninum* relied mainly on NLRC4 and ASC, as demonstrated for others pathogens (Silveira and Zamboni, 2010; Mascarenhas and Zamboni, 2017; Mascarenhas et al., 2017).

Several molecular and cellular events have been proposed as the trigger for NLRP3 inflammasome activation, including K^+^ efflux, Ca^2+^ signaling, reactive oxygen species (ROS), mitochondrial dysfunction, and lysosomal rupture (Sutterwala et al., 2014; Zamboni and Lima-Junior, 2015; He et al., 2016; Man et al., 2017; Ty et al., 2019). In general, ROS is known to cause inflammation in response to the destruction of tissues and release of danger signals (Ty et al., 2019). In this context, we found a crucial requirement of ROS to induce inflammasome activation and to control the parasite replication. Our data show that ROS is critical player for the activation of the inflammasome, while it is also a product of its activation, which is an important host defense mechanism against *N. caninum in vitro* and *in vivo*. Previous work, on different models, have shown that ROS are produced by NLRP3 activators, while are also essential secondary messengers for NLRP3 inflammasome activation (Reviewed by Martinon, 2010).

Although infections by different apicomplexan parasites have been shown to induce ROS, it is also relevant to the field to demonstrate that early sensing of *N. caninum* results in increased oxidative stress.

On the other hand, this positive inflammatory feedback loop between ROS and the inflammasome, if left uncontrolled, may lead to increased pathology in the infected hosts. This is one interesting topic that should be verified using proper experimental setups—different from those used in this work, that intended to verify initial host responses triggered by the infection. Classically, *N. caninum* is associated with reproductive disorders in cattle, with mid- to late-term abortions as its main clinical feature (Thilsted and Dubey, 1989). An increasing number of research groups have published promising results on *in vivo* and *ex vivo* bovine immune responses against *N. caninum* during gestation (Andrianarivo et al., 2001; Rosbottom et al., 2008; Rojo-Montejo et al., 2009; Bartley et al., 2013; Hecker et al., 2015; Pereyra et al., 2019). Also, some groups adopt the gestational mouse model in order to draw their experimental conclusions on this topic (Long and Baszler, 2000; Arranz-Solís et al., 2015; Aguado-Martínez et al., 2017). Regardless of the model, it is clear that IFN-γ is the crucial factor for parasite restriction, although its abundance in the placental environment is detrimental to fetal development (Innes, 2007). The association of active IFN-γ-dependent mechanisms and the inflammatory loop described here could be related to immunopathological features of the infection that are worth pursuing.

In conclusion, we show in this work that *N. caninum* induces activation of the NLRP3 and NLRC4 inflammasome complexes through a positive interplay with MyD88/NF-κB-dependent ROS production, that will lead to restriction of parasite replication by the hosts' immune system.

## Data Availability Statement

All datasets generated for this study are included in the article/Supplementary Material.

## Ethics Statement

The animal study was reviewed and approved by Ethics Commission on Animal Use (CEUA) from the Federal University of Uberlândia (UFU).

## Author Contributions

CM, DZ, and TM conceived and designed the experiments. CM, DL-J, FF, JA, and FS performed the experiments. CM, DL-J, PB, JM, and TM analyzed the data. JS, JM, DZ, and TM contributed reagents, materials, analysis tools and mice. CM and TM wrote the paper. All authors revised the manuscript.

## Conflict of Interest

The authors declare that the research was conducted in the absence of any commercial or financial relationships that could be construed as a potential conflict of interest.
