# Supplementary material for: Interplay Between Reactive Oxygen Species and the Inflammasome Are Crucial for Restriction of Neospora caninum Replication
Source: Front Cell Infect Microbiol. 2020 May 25;10:243. doi: 10.3389/fcimb.2020.00243 (PMC7261871; doi:10.3389/fcimb.2020.00243)

Supplementary material

Interplay between Reactive Oxygen Species and the Inflammasome are crucial for restriction of *Neospora caninum* replication

Caroline M. Mota[a], Djalma de S. Lima-Junior[b], Flávia Batista Ferreira França[a], Jhoan David Aguillón Torres[a], Patrício da Silva Cardoso Barros[a], Fernanda Maria Santiago[a], João Santana Silva[b], José Roberto Mineo[a], Dario S. Zamboni[b], Tiago W.P. Mineo[a]*

[a] Laboratory of Immunoparasitology "Dr. Mário Endsfeldz Camargo", Institute of Biomedical Sciences, Universidade Federal de Uberlândia, Uberlândia, Minas Gerais, Brazil.
[c] Departamento de Biologia Celular, Molecular e Bioagentes Patogênicos, Faculdade de Medicina de Ribeirão Preto, Universidade de São Paulo, Ribeirão Preto, São Paulo, Brazil.

* Corresponding author:
E-mail: tiago.mineo@ufu.br
Tel: +55 34 3225-8666
Postal Address: Laboratory of Immunoparasitology "Dr. Mário Endsfeldz Camargo", ICBIM/UFU, Av. Amazonas s/n, 4C01, 38405-320, Campus Umuarama, Uberlândia, MG, Brazil

Legends:

Supplementary Figure 1. The multiplicity infection (MOI; cell:parasite ratio) 0.5 of *N. caninum* represented a good ratio to assess inflammasome activation in marrow-derived macrophages (BMDMs). BMDMs were infected for 18 hours with *N. caninum* tachyzoites (NcLiv) in different MOI (0.5, 1 and 3) and IL-1β production was measured by ELISA (A). BMDMs were infected for 18 hours with *N. caninum* tachyzoites (NcLiv) in MOI 0.5 and parasite burden was determined by flow cytometry using DDAO-SE (fluorescent ester-based probe) stained tachyzoites, represented in % of DDAO-positive cells (B). Values are representative of two independent experiments and each condition was conducted at least in triplicates. Values indicating mean ± SEM of cytokine levels in relation the standard curve and fluorescence percentage (*$P < 0.05$; ANOVA with the Bonferroni multiple comparison post-hoc test or t test between naïve and *N. caninum* infected BMDMs).

Supplementary Figure 2. Different *Neospora caninum* isolates induced similar phenotypes in the inflammasome activation. BMDMs were infected for 18 hours with *N. caninum* tachyzoites (NcLiv or Nc-1; MOI 0.5). IL-1β production was measured by ELISA (A) and Caspase-1/11 activity was assessed by flow cytometry using fluorescent FAM-YVAD-FMK (FLICA) probe, represented in % of FLICA-positive cells (B). Values are representative of two independent experiments and each condition was conducted at least in triplicates. Values indicating mean ± SEM of cytokine levels in relation the standard curve and CASP-1/11 activity (*$P < 0.05$; ANOVA with the Bonferroni multiple comparison post-hoc test between naïve and *N. caninum* infected BMDMs).

Supplementary Figure 3. Comparison of the inflammasome activation by *N. caninum* and known agonists of the pathway. BMDMs were infected for 18 hours with *N. caninum* tachyzoites (NcLiv; MOI 0.5) or stimulated for 3 hours with 500 ng/mL LPS (TLR4/Caspase-11 agonist) and 2.5 μM ATP (NLRP3 inducer) for 1 hour. Caspase-1/11 activity was measured by fluorescent signal in a plate reader using fluorescent FAM-YVAD-FMK (FLICA) probe (A); IL-1β production was measured by ELISA (B); pore formation was quantified by propidium iodide (PI) incorporation (C); and ROS production was measured by the fluorescent DHCFDA probe (D). Values are representative of two independent experiments and each condition was conducted at least in triplicates. Values indicating mean ± SEM of cytokine levels in relation the standard curve and fluorescence levels (relative fluorescence units – RFU) (*$P < 0.05$; ANOVA with the Bonferroni multiple comparison post-hoc test between naïve and *N. caninum* infected BMDMs).

Supplementary Figure 1

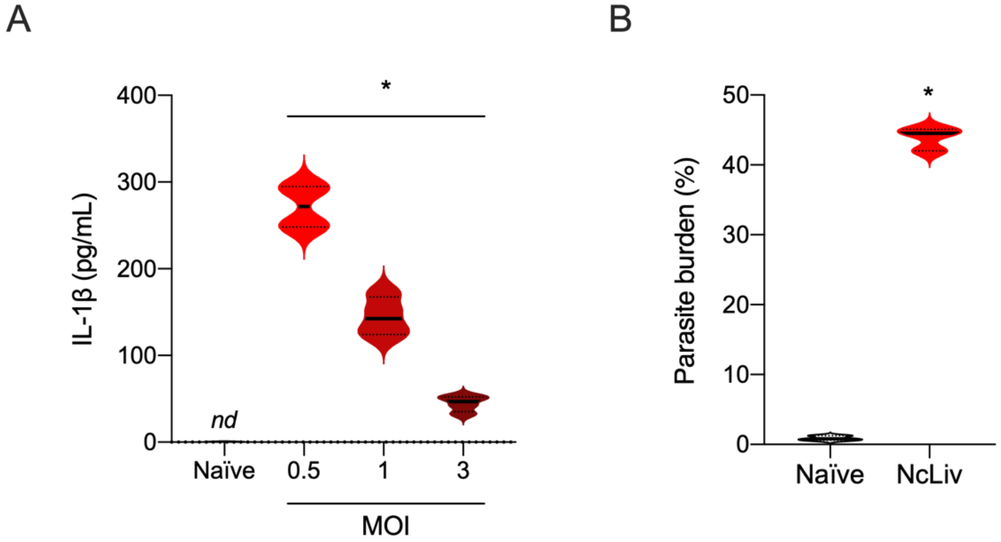

Supplementary Figure 2

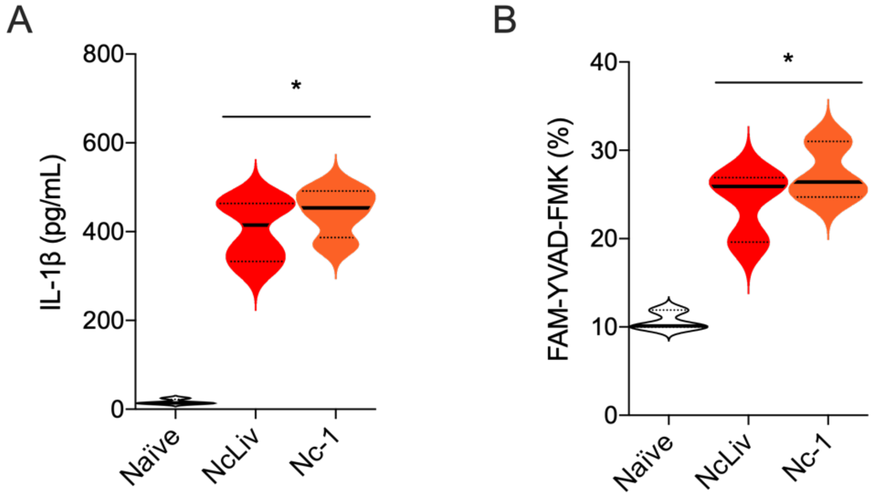

Supplementary Figure 3

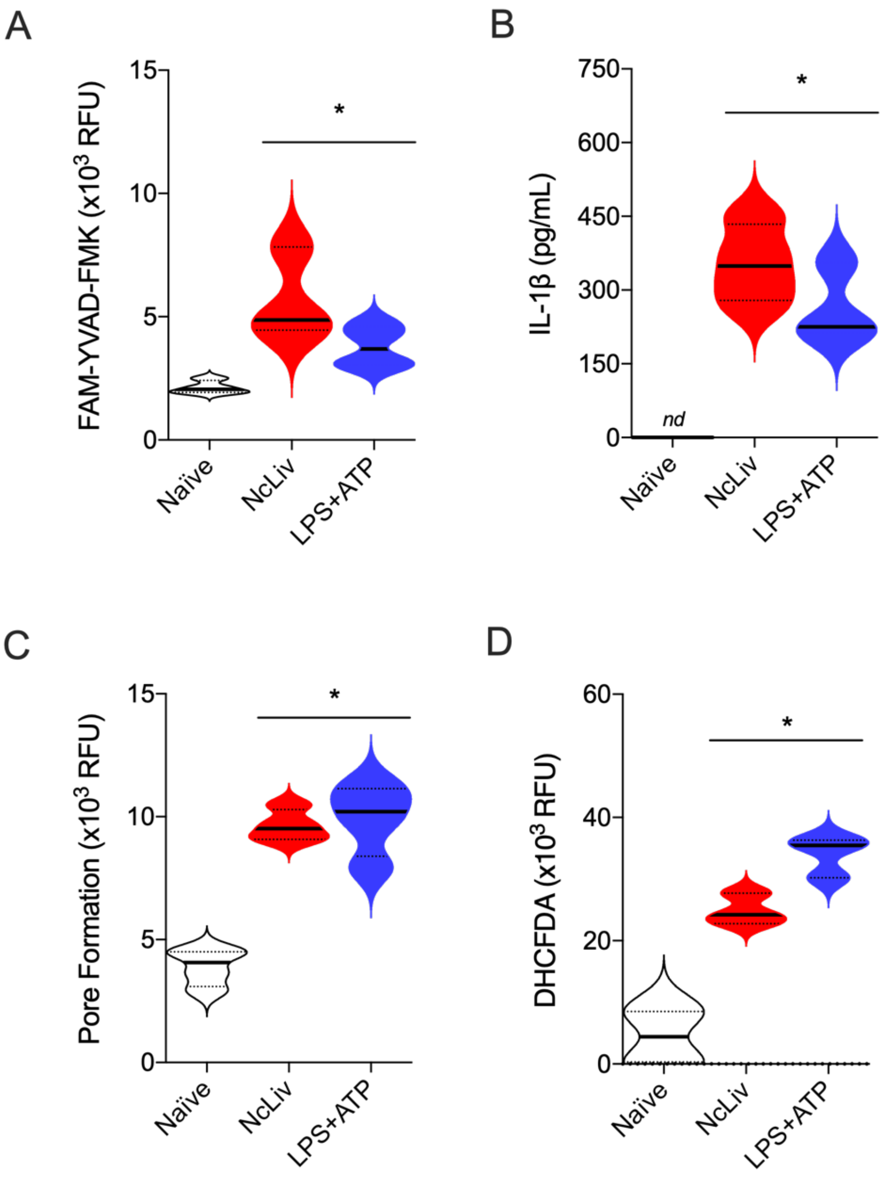

Supplement: Supplementary file 1 [file Data_Sheet_1.PDF]
